# The oldest fossil mushroom

**DOI:** 10.1371/journal.pone.0178327

**Published:** 2017-06-07

**Authors:** Sam W. Heads, Andrew N. Miller, J. Leland Crane, M. Jared Thomas, Danielle M. Ruffatto, Andrew S. Methven, Daniel B. Raudabaugh, Yinan Wang

**Affiliations:** 1 Illinois Natural History Survey, Prairie Research Institute, University of Illinois at Urbana-Champaign, Champaign, Illinois, United States of America; 2 Department of Biology, Savannah State University, Savannah, Georgia, United States of America; 3 Department of Plant Biology, University of Illinois at Urbana-Champaign, Urbana, Illinois, United States of America; 4 Independent Researcher, Arlington, Virginia, United States of America; Institute of Botany, CHINA

## Abstract

A new fossil mushroom is described and illustrated from the Lower Cretaceous Crato Formation of northeast Brazil. *Gondwanagaricites magnificus* gen. et sp. nov. is remarkable for its exceptional preservation as a mineralized replacement in laminated limestone, as all other fossil mushrooms are known from amber inclusions. *Gondwanagaricites* represents the oldest fossil mushroom to date and the first fossil mushroom from Gondwana.

## Introduction

Exceptionally preserved fossils can shed important and unprecedented light on the history of life. Particularly remarkable deposits, known as Lagerstätten, yield fossils characterized by preservation of soft tissues that decay rapidly and which are not normally preserved. In many cases, large and important groups of soft-bodied organisms would be missing entirely from the fossil record if not for their exceptional preservation in Lagerstätten. Mushrooms, an ecologically important group of fungi in the order Agaricales, produce fleshy, gilled fruiting bodies (called basidiomes) that are rarely fossilized [1]. While certainly ancient, they have an extremely depauperate fossil record with only ten fossil mushrooms reported to date, all unique amber inclusions ranging from mid-Cretaceous to Early Miocene in age [2–8]. Here we report the discovery of a new fossil mushroom that is unique in its preservation as a mineralized replacement, and the oldest yet encountered. The specimen comes from the laminated limestones of the Crato Formation, which outcrop on the northern flanks of the Chapada do Araripe in Ceará, Brazil; a Lagerstätte famous for the exceptional preservation of its diverse Lower Cretaceous paleobiota [9–11].

## Material and methods

The specimen comprises a single, nearly complete mushroom preserved as a primarily goethitic replacement on a small slab (approximately 50 × 60 mm) of typical, buff-colored, millimetrically-laminated limestone from the Nova Olinda Member; the lowermost unit of the Crato Formation. It is housed in the URM Herbarium at the Universidade Federal de Pernambuco in Recife, Brazil, having been repatriated from the Illinois Natural History Survey Paleontological Collection. It was studied using a Zeiss SteREO Discovery.V20 zoom stereomicroscope with a Plan-Apochromat S 0.63x f/ Reo WD = 81 mm objective. Photographs were taken using a Canon 5D Mark III and MP-E 65 mm macro lens mounted to a Cognisys Stackshot motor rail on a copy stand. Multiple high-resolution images were then stacked using HeliconSoft’s Helicon Focus 6 and subsequently stitched together as a mosaic using Photoshop CC. Scanning electron micrographs were produced using a JEOL JSM-6060LV SEM.

### Nomenclature

The electronic version of this article in Portable Document Format (PDF) in a work with an ISSN or ISBN will represent a published work according to the International Code of Nomenclature for algae, fungi, and plants, and hence the new names contained in the electronic publication of a PLOS ONE article are effectively published under that Code from the electronic edition alone, so there is no longer any need to provide printed copies. In addition, new names contained in this work have been submitted to MycoBank from where they will be made available to the Global Names Index. The unique MycoBank number can be resolved and the associated information viewed through any standard web browser by appending the MycoBank number contained in this publication to the prefix http://mycobank.org/MB/. The online version of this work is archived and available from the following digital repositories: PubMed Central, LOCKSS.

## Results

### Systematic paleontology

Kingdom Fungi (L.) Moore, 1980; Phylum Basidiomycota Moore, 1980; Class Agaricomycetes Doweld, 2001; Order Agaricales Underwood, 1899; Family *incertae sedis*

#### *Gondwanagaricites magnificus* Heads, A.N. Mill. et J.L. Crane, gen. et sp. nov.

(Figs 1 and 2)

**Fig 1:**
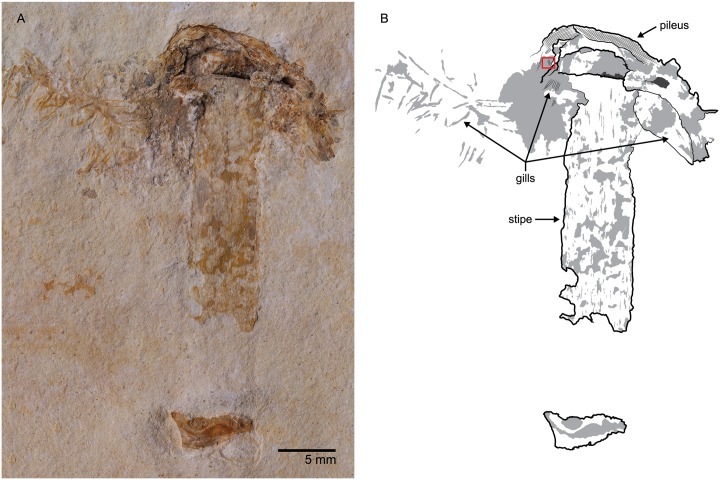
*Gondwanagaricites magnificus* gen. et sp. nov. (*A*) Photomicrograph of holotype (URM 88000) showing general habitus. (*B*) Interpretive drawing of (*A*) with major morphological features indicated. The red box indicates the position of gills shown in Fig 2.

**Fig 2:**
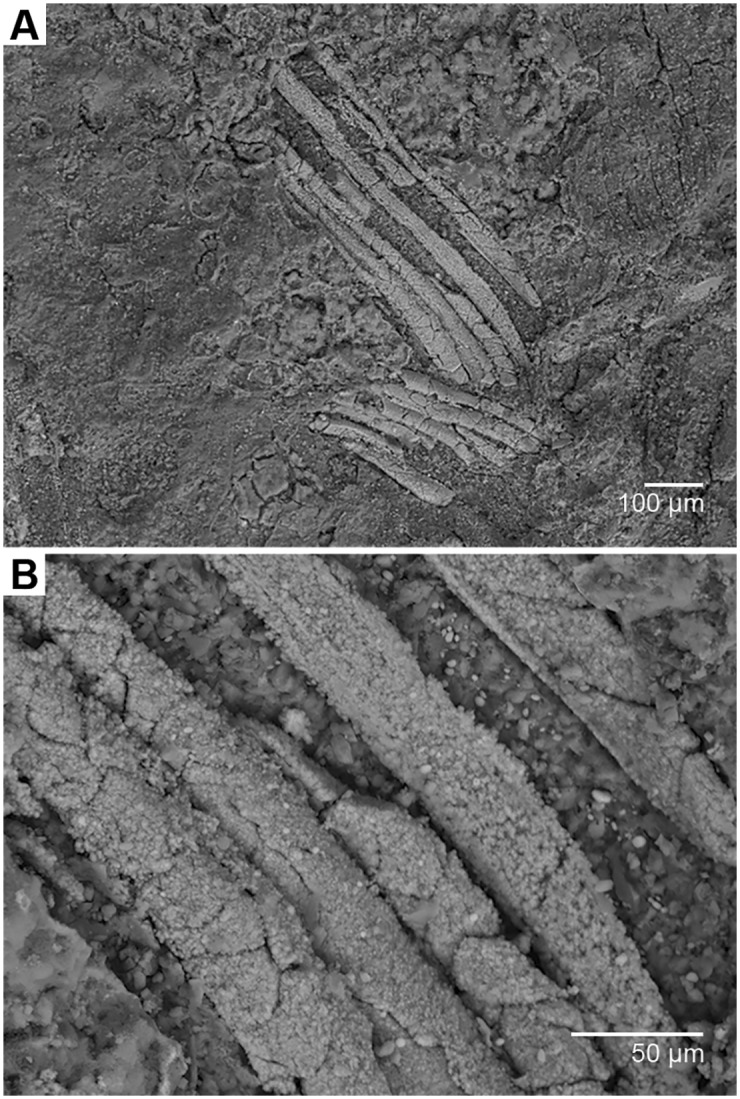
Scanning electron micrographs of the gills of *Gondwanagaricites magnificus* gen. et sp. nov. (*A*) Section of preserved gills (location indicated by red box on Fig 1B). (*B*) close-up view of (*A*) showing detailed structure.

[urn:lsid:mycobank.org:names:MB821206]

#### Holotype

Brazil: Ceará: Chapada do Araripe; Crato Formation: Nova Olinda Member (Lower Cretaceous: Upper Aptian, 113–120 Ma); URM-88000. While precise locality details are unknown, the lithology of the matrix is consistent with the specimen having been collected in one of the extensive quarry complexes near the town of Nova Olinda (7.0939°S, 39.6796°W).

#### Etymology

The genus name is a combination of Gondwana, the ancient supercontinent, the Greek word *agarikon*, “a mushroom,” and the Greek suffix *-ites*, denoting a fossil. The specific epithet is the Latin adjective *magnificus*, meaning “magnificent” or “splendid” in reference to the remarkable preservation of the holotype.

#### Description

Basidiome color unknown (preserved as orange-brown goethitic replacement). Pileus 10.0 mm diameter, 7.5 mm high at widest point; apparently circular, convex; probably glabrous and striate; margin slightly incurved; veil absent; context 3.0 mm thick. Lamellae (gills) 4.5 mm broad at widest point, broadly attached to stipe apex; edge entire, up to 50μm wide. Stipe 34.0 mm long, 6.5 mm wide, straight, cylindrical, with longitudinal striations, annulus absent, base slightly bulbous. Basidiospores not observed.

#### Comments

While *Gondwanagaricites* is without doubt a gilled mushroom in the Agaricales, familial placement is presently impossible since no evidence of basidiospores was found during SEM examination of the specimen. The general habitus of *Gondwanagaricites* is reminiscent of mushrooms in the family Strophariaceae and placement in this family would be supported by the small size and robust shape of the overall basidiome, the thick context of the pileus, the putative complete attachment of the gills to the central stipe, and the apparent absence of a universal and partial veil. However, a number of other mushroom families present similar basidiome morphology (e.g., Agaricaceae, Tricholomataceae, Bolbitiaceae, etc.) and can only be separated by detailed studies of basidiospore shape, ornamentation, and coloration. Thus, since the spores of *Gondwanagaricites* were not observed, we refrain from assigning the new genus to a family.

## Discussion

Fungi are ecologically diverse, geographically widespread, speciose organisms that account for the second largest group of eukaryotes [12]. Despite their global distribution and evolutionary history extending some 1,430 Ma [13], the fossil record for fungal structures other than spores is exceedingly scant with reports of mostly sexual [14–18] and asexual stages [19–23] of ascomycetes from mid-Cretaceous to Miocene ambers. The Basidiomycota contains over 30,000 extant species [24], but their fossil record—especially in the case of gilled mushrooms—is nearly non-existent due to their ephemeral nature and a strong preservational bias against their fleshy basidiomes [1–8]. The earliest report of a member of the Basidiomycota is from hyphae with diagnostic clamp connections dating *c*. 330 Ma from the Upper Visean (Mississippian) of France [25]. Only ten fossils resembling modern-day gilled mushrooms have been recorded to date, all from amber. The hitherto oldest fossil mushroom, *Palaeoagaricites antiquus*, was reported from mid-Cretaceous Burmese amber (*c*. 99 Ma) [6]. More recently, four unnamed mushrooms placed in the Agaricales have also been reported from Burmese amber [8]. *Archaeomarasmius leggetti* was recorded from Cretaceous amber (*c*. 90–94 Ma) from New Jersey, USA [3,4]. Most recently, *Gerontomyces lepidotus* was reported from Eocene Baltic amber (*c*. 45–55 Ma) from the Samland Peninsula of Russia [7]. Three other mushrooms, *Aureofungus yaniguaensis* [5], *Coprinites dominicana* [2], and *Protomycena electra* [3,4] have all been recorded from Early Miocene amber (Burdigalian, *c*. 16–18 Ma) from the Dominican Republic.

*Gondwanagaricites magnificus* represents the oldest fossil record of a gilled mushroom and is the only fossil mushroom known from a mineralized replacement. The unique specimen extends the geological range of gilled mushrooms back by approximately 14–21 million years and confirms their presence in Gondwana during the Early Cretaceous. Molecular clock estimates suggest the divergence of the Basidiomycota around 500 Ma to 1.2 billion years [26] and *G*. *magnificus* establishes the earliest calibration point so far for the Agaricales, with a new minimum age of 113–120 Ma.
