# Correction: The oldest fossil mushroom

**DOI:** 10.1371/journal.pone.0199660

**Published:** 2018-06-21

**Authors:** The PLOS ONE Editors

Following publication of this article [1], the authors contacted *PLOS ONE* to raise to our attention concerns that the genus and species names (*Gondwanagaricites* and *Gondwanagaricites magnificus*, respectively) did not meet the requirements of Articles 42.1, 35.1, and 43.3 under the International Code of Nomenclature for Algae, Fungi, and Plants. Specifically, a MycoBank number for the genus was not included in the paper, and the illustration depicting the holotype (Fig 1A) was not specifically identified.

The genus and species names have now been published in a Mycological Progress article [2], in which new MycoBank numbers are assigned:

*Gondwanagaricites* Heads, A.N. Mill. et J.L. Crane, gen. nov. MB821896

*Gondwanagaricites magnificus* Heads, A.N. Mill. et J.L. Crane, sp. nov. MB821897

The *PLOS ONE* Editors regret a delay in issuing a Correction to the *PLOS ONE* article and we ask readers to refer to the Mycological Progress article [2] when referring to the species name and MycoBank numbers.
